# Mapping the Global Landscape of Long-Term Care Insurance Research: A Scientometric Analysis

**DOI:** 10.3390/ijerph19127425

**Published:** 2022-06-17

**Authors:** Long Xia, Lulu Chai, Hanyu Zhang, Zhaohui Sun

**Affiliations:** Department of Law and Political Science, North China Electric Power University, Baoding 071003, China; 51951261@ncepu.edu.cn (L.X.); 18437923011@163.com (L.C.); zhy2001529@126.com (H.Z.)

**Keywords:** long-term care insurance, scientometric review, CiteSpace, visualization

## Abstract

With the aging population increasing dramatically and the high cost of long-term care (LTC), long-term care insurance (LTCI) has expanded rapidly across the world. This review aims to summarize the status quo, evolution trends, and new frontiers of global LTCI research between 1984 and 2021 through a comprehensive retrospective analysis. A total of 1568 articles retrieved from the Web of Science Core Collection database were systematically analyzed using CiteSpace visualization software (CiteSpace 5.8. R2, developed by Dr. Chaomei Chen at Drexel University (Philadelphia, PA, USA)). The overall characteristics analysis showed that LTCI is an emerging research field in a rapid development stage—nearly 50% of articles were published in the past five years. The most productive LTCI research institutions and authors are located primarily in Japan and the USA. A rigorous analysis based on a dual perspective of references and keywords was applied to reveal that common LTCI hot topics include disability in the elderly, LTC financing, demand for and supply of LTCI, and LTCI systems. In addition, LTCI research trends have shifted from the supply side to the demand side, and from basic studies to practical applications. The new research frontiers are frailty in the elderly, demand for LTCI, and LTCI systems. These findings can provide help and reference for public health practitioners and researchers, as well as help with the sustainable development of LTCI research.

## 1. Introduction

In recent years, the world’s aging population has shown a trend of rapid increase. According to World Population Prospects (2020 revision), the number of people aged 60 and older was 1.05 billion in 2020, accounting for 13.46% of the world’s total population. It is estimated that this number will reach 1.41 billion (16.46%) in 2030 and exceed 2 billion (21%) in 2050. The aging problem has become a great worldwide issue, especially in developed countries. Japan is the most aged country in the world—34.32% of its population is aged 60 or older. Germany, Canada, and the USA are also confronted with severe aging populations, with a proportion of older people of 28.63%, 24.89%, and 22.88%, respectively. This increase has led to a growing number of disabled elderly, creating a huge need for long-term care (LTC), which places a heavy economic burden on their families. In order to resolve this problem, long-term care insurance (LTCI) has been explored and developed. LTCI refers to the reimbursement of nursing expenses for people with chronic diseases or functional impairments over a relatively long period of time. Germany was the first country to implement public LTCI in 1994 and its coverage has reached about 90% [1]. Japan and South Korea followed. The LTCI in Japan covers senior citizens aged 65 or older and those aged 40–64 with age-related diseases. The USA is a typical country that carries out private LTCI. By the end of 2018, the premium income of LTCI in the United States reached 12.778 billion, covering nearly 7 million people. The experience of developed countries over the past 20 years shows that LTCI can not only reduce the risk caused by the expansion of the disabled elderly population but also expand state welfare and finance home care and nursing home care for the frail elderly [2]. Recently, some developing countries have also experimented with LTCI. In 2016, China set up 15 pilot cities of LTCI, and increased 14 more pilot cities, including Shijingshan District, Beijing, in September 2020.

As the importance of developing LTCI has been realized by an increasing number of countries, research on LTCI has experienced significant growth over the past two decades. Studies have shown that LTCI is a multidisciplinary scientific research area involving geriatrics and gerontology [2,3]; economics [4,5,6]; healthcare sciences and services [7,8]; public, environmental, and occupational health [3,9]; health policy and services [10,11,12]; nursing [13,14,15]; psychiatry [9,10], including the demand for and supply of LTCI [10,15,16,17,18]; LTCI expenditure and financing [6]; and the establishment of LTCI systems [16,19]. To an extent, the combination of expertise in various aspects has contributed to the fragmented nature of LTCI research. A systematic overview of LTCI field is needed. However, some recent review studies have only addressed the specific dimensions of LTCI. For example, Houde and Gautam [20] reviewed the LTCI program in Japan and the present payment system of LTC services in the USA. Eling and Ghavibazoo [21] carried out a research review on three major research areas of LTCI: financing, demand, and insurability. Chen et al. [22] presented a review of the development of the public LTCI system in four respects, comprising a comparison of public LTCI systems in different countries, the influence and the challenge of public LTCI, and the relationship between public and private LTCI. In sum, previous review papers on LTCI have either adopted a qualitative approach or focused on a specific aspect of LTCI. As a result, there is still no comprehensive and in-depth review of the published research, which makes it difficult to accurately understand the evolution of the LTCI field, identify the research contributions and gaps, and establish future research agendas.

In this paper, we aimed to provide an overview of the most influential scientific literature published on Web of Science in a quantitative way to better understand the LTCI field. A comprehensive scientometric analysis and substantial discussion of research progress in LTCI were provided to: (1) summarize significant publication patterns in LTCI with basic statistics and advanced analytics; (2) evaluate research performance from multiple perspectives, such as countries/regions, institutes, and authors; (3) and present the research foci, trends, and frontiers of LTCI from references and keywords. The results provide evidence of the current status and future trends in LTCI, which helps scholars to understand the panorama of this topic and foresee the dynamic directions of this field of research.

## 2. Materials and Methods

### 2.1. Scientometric Analysis in CiteSpace

CiteSpace version 5.8. R2 was selected to conduct scientometric analyses. This software was first developed by Dr. Chaomei Chen. It is capable of visualizing burst terms and betweenness centrality to identify emerging trends, turning points, and the internal relations among different research fronts. Furthermore, it embodies eight different visualization graphs to represent the patterns of the scientific literature, such as cluster views and timeline views. Nodes and links are the building blocks of visual maps created by CiteSpace. The types of nodes include authors, institutions, countries, keywords, and cited references. According to Chen [23], nodes are made up of “tree rings” of different colors, of which red rings denote a citation burst and purple rings indicate the degree of a node’s betweenness centrality. In addition, links represent the co-occurrence or co-citation relationship between nodes. For example, the color of a link indicates the year of the first relationship established between two nodes, and the thicker the link, the more cooperation between the two nodes.

CiteSpace supports several types of bibliometric studies, including collaboration network analysis, document co-citation analysis, keyword co-occurrence analysis, and author co-citation analysis. In this paper, we mainly focus on the first three bibliographic techniques. Collaboration network analysis is critical to understanding scholarly communication and knowledge diffusion [24]. It evaluates the published contributions and academic impact of authors, countries, and institutions through a visual network of scientific collaboration [23]. Both document co-citation and keyword co-occurrence analysis are based on co-occurrence analysis techniques, which are used to measure the frequency of co-occurrence of keywords and cited documents in the same document. Document co-citation analysis provides insights into the intellectual structures of a knowledge domain and identifies the quantity and authority of references cited by publications [24]. Keyword co-occurrence analysis tends to be employed to explore changes in research themes in a research field by measuring the frequency of pairs of items occurring in the entire body of literature in a selected field. In addition, users can also specify time slicing, set up thresholds, select pruning and merging approaches, and conduct visual inspection (Fu et al. [25]).

### 2.2. Data Collection

**Figure 1:**
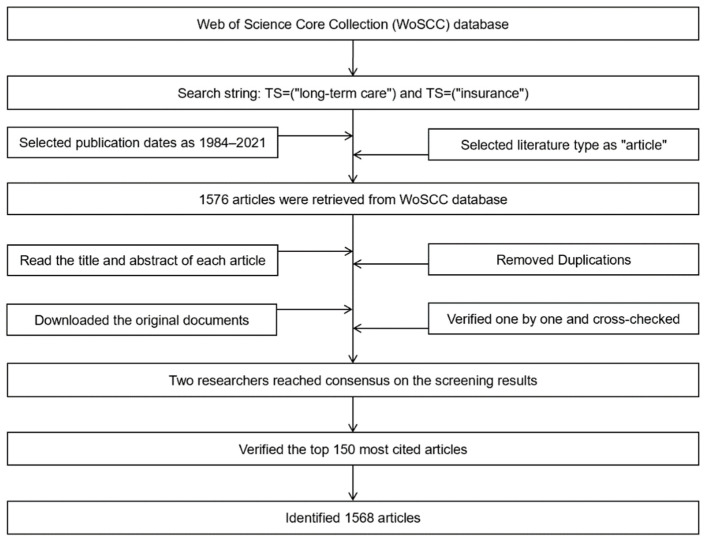
The process of data collection.

The data used in this article are from the Science Citation Index Expanded (SCI-E) and Social Sciences Citation Index (SSCI) in the Thomson Reuters Web of Science Core Collection (WoSCC) database, which is one of the world’s leading citation databases with multidisciplinary coverage of over 10,000 high-impact journals in the sciences, social sciences, and arts and humanities. The literature retrieval was carried out independently by 2 researchers simultaneously on 31 December 2021. The retrieval conditions were as follows: TS = (‘‘long-term care’’) and TS = (‘‘insurance’’). We performed a topic search of all publications that contained these words in title, abstract, and keywords. Meanwhile, the publication dates were set from 1984 to 2021 and literature type of article was chosen. After retrieving, the title and abstract of the obtained literature were read to determine whether they were relevant to the research topic, and eight repeated documents were removed. Then, the original texts of all the documents were downloaded, verified one by one and cross-checked. If there were differences and they were difficult to determine, the group members would study and discuss together, or the decision would be made by the third researcher. According to our retrieval results, the two researchers reached the same conclusion on the screening results, and the further validity test showed that the top 150 most cited articles were closely related to LTCI, indicating that our retrieval strategies and search terms were appropriate. The data collection process is shown in Figure 1. Finally, a total of 1568 literature results published between 1984 and 2021 were retrieved, each of which included the title, author, abstract, keywords, references, and other information.

## 3. Results and Discussion

### 3.1. Current Status of LTCI Research

**Figure 2:**
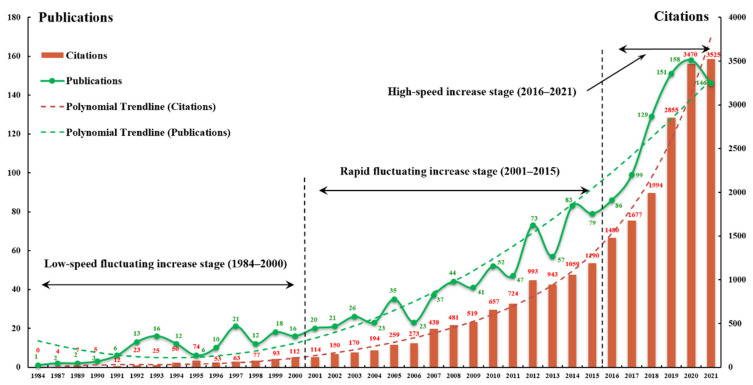
The annual number of publications and citations in LTCI field from 1984 to 2021.

The 1568 journal publications were published in six languages. The vast majority of these articles were written in English (1457 records, accounting for 92.92%), followed by German (92, 5.87%). Korean (12), French (3), Spanish (3), and Japanese (1) were also contributing languages, although their shares were all below 1%. English remained the dominant language, which is to be expected given that most journals indexed by WoSCC were published in English. These publications examined LTCI from multiple perspectives, with 793 conducted from the health and medical care disciplines, accounting for 50.57%. Publications from economic and financial disciplines take second place (297, 18.94%). There are also publications based on the perspectives of the health policy services (179, 11.42%), sociology (60, 3.83%), demography (25, 1.60%), and psychology (24, 1.53%). Figure 2 presents the current status of LTCI research by the annual distribution of publications and citations between 1984 and 2021. The green points represent the number of publications per year, and the bar graphs illustrate the annual citation counts. Additionally, two dotted lines show the trends that were identified by fitting a polynomial to the data. According to Figure 2, distribution was divided into three stages, as follows:

1. Low-speed fluctuating increase stage (1984–2000): It can be seen from Figure 2 that fewer than 10 articles on the topic of LTCI were published before 1990. There were no articles in the years 1985, 1986, and 1988, so they are not shown here. Since the first article was published in 1984, the LTCI research obtained a very slow and fluctuant increase in the following 16 years. Notably, 1997 was the most productive year, with 21 articles, followed by a certain degree of decline, and then a slow increase. In terms of annual citation counts, there was no citation of LTCI literature in 1984, and fewer than 10 citations before 1990. The number has increased steadily over time since 1991. The average cited references increased from 1.67 in 1990 to 7 in 2000, demonstrating that the influence of the published literature was improved. Overall, except for the year 2000, numbers of citations in other years were all below 100, which might be due to the limited amount of published literature in those years.
2. Rapid fluctuating increase stage (2001–2015): Over this period, the number of articles began to grow continuously in a wave-like manner and reached its peak in 2014, with 83 records. Moreover, the results of citations agreed with the publication trends, indicating that more high-quality papers had been published. Articles published in 2014 and 2015 received more than 1000 citations. Moreover, two annual citation bursts were found in 2007 and 2012, increasing by 165 and 269 over the previous year, respectively. The increasing trend during this period is partially attributed to the greater availability of online documents and more convenient access for researchers, as well as the implementation of LTCI by an increasing number of countries, which attracted much attention from scholars.
3. High-speed increase stage (2016–2021): The annual number of publications increased dramatically from 2016. Furthermore, the overall publication percentage during this stage accounted for 49.04% of all publications. These results suggest that LTCI is an emerging research field in a rapid development stage. Through curve fitting based on the data of the last six years, the number of publications was estimated to reach 142 in 2022 and 125 in 2023, indicating that the LTCI research output will continue to stay at a relatively high level in the next few years, although it presents a declining trend. The trend line of the annual numbers of citations showed that there was an explosive growth in 2019, and the numbers all exceeded 2800 in the past three years, which implies a wider influence of LTCI research in recent years. Building on many breakthroughs in the study period 2001–2021, especially in the past six years, LTCI has become one of the most important and dynamic fields of population aging research.

### 3.2. Major Contributors to LTCI Field

LTCI is a research field that has been frequently approached by many different countries, institutions, and authors, but the identity of the major contributors still remains unknown. In this section, we attempt to answer this question by conducting country collaboration analysis and institution collaboration analysis via CiteSpace and extracting productive authors from the WoSCC database.

#### 3.2.1. Leading Country Analysis

**Figure 3:**
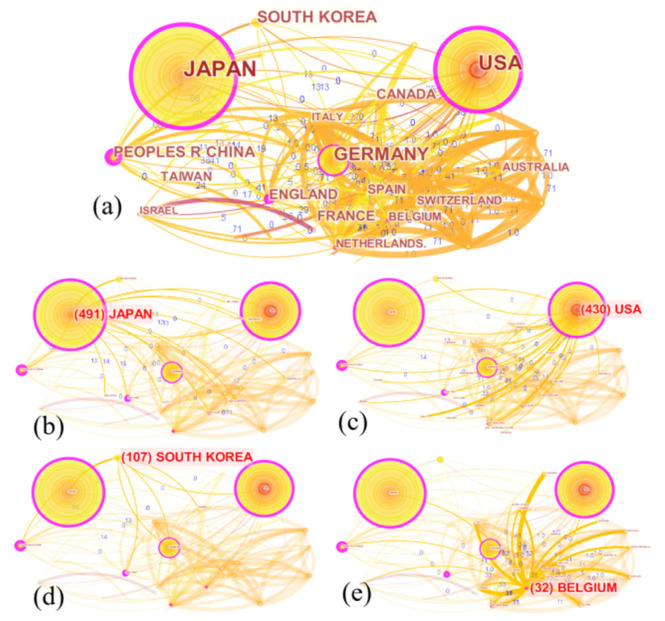
A visualization of the country collaboration network: (**a**) all countries/regions; (**b**) Japan; (**c**) USA; (**d**) South Korea; (**e**) Belgium.

The map of geographical global distribution of LTCI research was generated by CiteSpace using the following parameters: (1) time slice from 1984 to 2021; (2) years per slice = 1; (3) term source = title/abstract/author keywords/keywords plus; (4) node type = country; (5) pruning = none; (6) select top 50 most cited articles per slice. As shown in Figure 3, the research network includes 55 nodes and 209 links. The nodes represent the “countries”, and the links between them indicate the collaborative relations. It is worth noting that the size of nodes was proportional to the publication volume, while the thickness of the connecting lines between countries demonstrated the intensity of cooperation. Furthermore, the purple circles demonstrate key countries with high betweenness centrality above 0.1.

In order to obtain more information about countries/regions, the top 15 contributors ranked by publication counts and centrality are shown in detail in Table 1, including seven European countries, five Asian countries, two North American countries, and one Oceanian country. Additionally, among them, Japan ranked first by contributing 491 publications, followed by the USA with 430 articles and Germany with 201 articles. In terms of the large number of publications, it seems that these three contributors form a leading LTCI research group. As is well known, LTCI originated in developed countries and gradually gained maturity, attracting a large number of scholars to conduct research in its theories and applications [26,27]. Therefore, this research was dominated by developed countries. It is notable that China was the only developing country (Taiwan Province of China is treated as a developed region) in the top 15 most productive countries. This may be mainly due to the rapid expansion of China’s LTCI pilot program, which has aroused great interest among scholars in their research.

As shown in Table 1, the top seven countries in terms of betweenness centrality (purple rings) were the USA (0.39), Japan (0.24), China (0.24), England (0.22), Germany (0.15), Belgium (0.19), and the Netherlands (0.13). The collaboration networks of four countries are shown in Figure 3b–e. Japan and the USA had dense network structures, representing the symbolic significance of these two countries for LTCI research and the pivotal role of links, which play an intermediary role for those nodes that cannot be directly connected. South Korea ranked fourth in publication counts, but it had few links with other countries, with a betweenness centrality of less than 0.01, demonstrating that it did not actively participate in collaborative research activities. However, Belgium, though it had only 32 publications, worked closely with other countries and had a significant international influence in the field of LTCI. Therefore, for some countries with sparse network structures, such as North Korea, France, and Canada, their international academic influence should be enhanced with stronger collaborations. Furthermore, globalization requires cooperation and looking into research issues from multiple perspectives. However, the existing national cooperation networks suggest that this field still has strong potential for international cooperative network development.

#### 3.2.2. High-Yielding Institution Analysis

**Figure 4:**
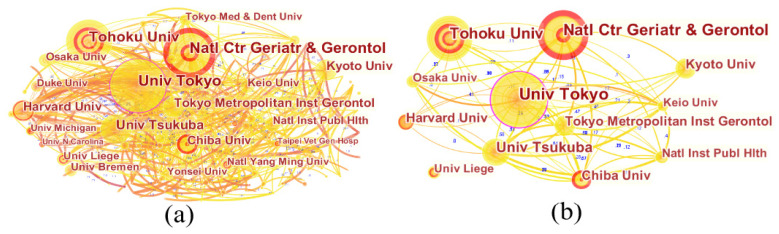
A visualization of the institution collaboration network: (**a**) all institutions; (**b**) top 15 productive institutions.

The institution map generated by CiteSpace kept the same parameters, except for the node type being changed from “country” to “institution”. The network consisting of 638 nodes and 870 links is depicted in Figure 4. As is shown, the most influential institutions focusing on LTCI are mainly from Japan. Table 2 lists the top 12 institutions with the greatest outputs in this area, with a contribution to 455 articles, accounting for 29.58% of the total. Within these 12 institutions, there were 10 Japanese institutions, once again indicating that Japan had significant LTCI research capabilities. Specifically, the University of Tokyo had the greatest number of publications, with a total of 78 papers, accounting for 5.07% of all publications. In second position was the National Center for Geriatrics and Gerontology with 64 publications (4.16%), followed by Tohoku University (64, 4.16%) and the University of Tsukuba (43, 2.80%). One prominent institution in the USA was Harvard University, with 31 publications (2.02%), which ranked fifth. Moreover, one European institution, the University of Liege (18, 1.17%), also made a significant contribution to this research.

From the perspective of institutional cooperation, the density of the network was 0.0043, indicating that the cooperation intensity between research institutions was weak, and a wide and close cooperation network had not been formed. The collaboration network of 12 highly productive institutions is mapped in Figure 4b. It can be seen from the figure that, except for the University of Liège, the other 11 institutes had evident collaborations with one another. However, though Harvard University cooperated with several Japanese institutions, the cooperation relationships between them were not close, with the link strengths all below 0.2. It is indicated that the existing cooperation mainly focused on the collaboration of domestic institutions, while the cooperation between international research institutions needs to be strengthened. Moreover, institutions with strong bursts (nodes with red inner rings) reflected significant increases in publications over short periods of time. In terms of the burst of publications, Chiba University ranked first, with a burst value of 7.76 (2018–2021), followed by National Center for Geriatrics and Gerontology (6.88, 2017–2021), indicating that these institutions have grown significantly in LTCI research in recent years.

#### 3.2.3. Productive Author Analysis

**Figure 5:**
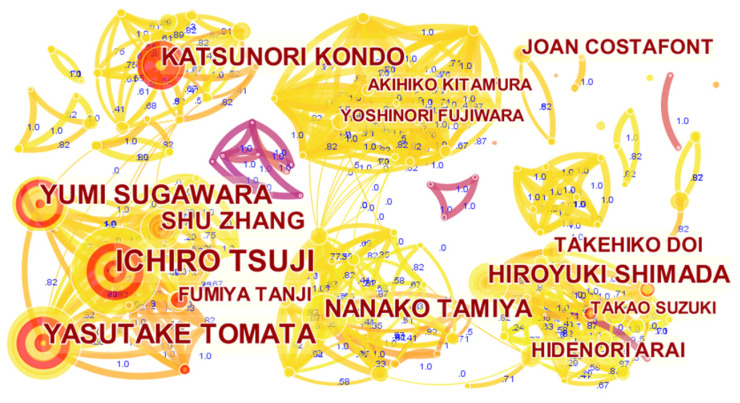
A visualization of the author collaboration network.

According to the collaboration networks of authors, it seems that the authors tended to collaborate with a single, highly productive author, thus forming co-author clusters. As shown in Figure 5, there are five main co-author clusters, with Tsuji, Kondo, Fujiwara, Tamiya, and Shimada as the central authors, respectively. For example, most of the authors in the Tsuji cluster were from Tohoku University, and they paid close attention to empirical research through the Ohsaki Cohort 2006 Study; the Kondo cluster used the Aichi Gerontological Evaluation Study to examine the influential factors of LTCI needs and costs; the Fujiwara cluster mainly conducted research on preventing frailty in the elderly; the Tamiya cluster had a common focus on the improvement of the LTCI system in Japan; the Shimada cluster applied a prospective cohort study to examine factors that increase the risk of disability in the elderly. However, all authors had low centrality (<0.1), revealing that the central authors did not occupy the position of “intermediaries”. The trend of LTCI development requires multi-dimensional cooperation across disciplines and institutions; hence, these authors could have the potential to become a “bridge” connecting institutional cooperation in the future, and strive to build a more dynamic and open cooperative network.

In terms of the most influential researchers on LTCI, Tsuji ranked at the top of the list. Tsuji is a well-known professor at Tohoku University, and a highly representative scholar in life sciences, healthcare management, medical sociology, and public health. Over the past 10 years, Professor Tsuji has been devoted to the Ohsaki Cohort 2006 Study, a novel population-based prospective cohort study, which is composed of people aged 65 years and older in Japan, who were living in Ohsaki City, on 1 December 2006 [28]. The five notable articles from Tsuji show that he conducted a series of studies on the effects of factors such as green tea consumption, dietary patterns, earthquakes, and tsunamis on physical and mental disability status which were evaluated by the LTCI certification status (see Table 3).

### 3.3. Intellectual Landscape of LTCI Field

In this section, we describe the intellectual landscape to identify hot topics, research frontiers, and evolution trends in LTCI research based on an analysis of cited references and co-occurring keywords. The analyses are described below.

#### 3.3.1. Document Co-Citation Analysis

**Figure 6:**
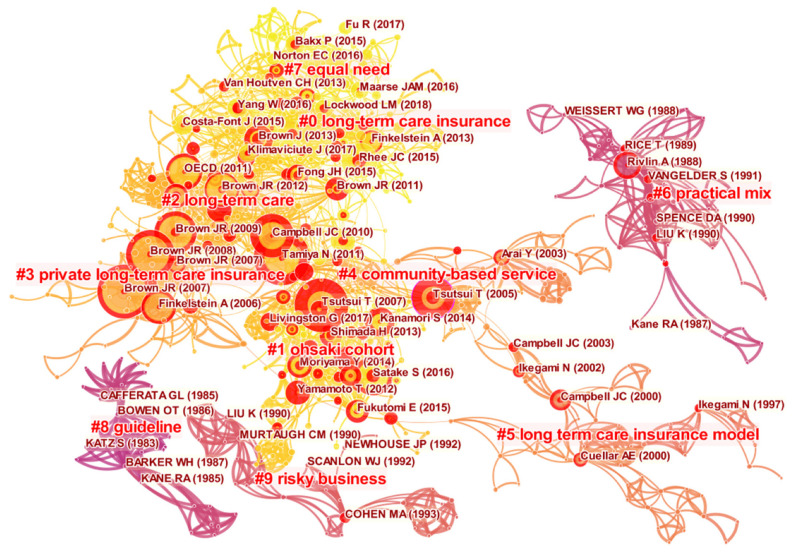
A visualization of the document co-citation network in LTCI research. Note: # represents a knowledge cluster.

We conducted document co-citation analysis to analyze the underlying intellectual structures of the LTCI domain and disclose its knowledge roots. In this process, co-citation clusters were also identified, which could reflect the evolution process of the scientific activity of this field. The following parameters in CiteSpace were used: (1) time slice from 1984 to 2021; (2) years per slice = 1; (3) term source = title/abstract/author keywords/keywords plus; (4) node type = reference; (5) pruning = none; (6) select top 50 most cited articles per slice. After running CiteSpace, a co-citation cluster network, which contained 1244 references and 3594 links, was visualized. Its modularity Q and mean silhouette were 0.8732 and 0.9132, respectively, suggesting a good inter-cluster connection within the network and considerable partition of the network. A total of 185 co-citation clusters were identified from the co-citation network using the log-likelihood ratio (LLR) algorithm. Each cluster was a group of tightly coupled references representing a thematic concentration in the bibliographic landscapes. Moreover, its size was measured by the number of references and sequenced in descending order of the cluster numbering. As shown in Figure 6, we focused on the top 10 largest clusters in this study.

Generally, the intellectual base of a field is composed of a group of literature works with high betweenness centrality, high citation, or strong bursts. Table 4 lists the top 15 most highly cited references with citation frequency of over 13 times (i.e., No. 1 to No. 15) based on the above three features, distributing in six clusters (i.e., #0–#4 and #6). In terms of high betweenness centrality, one reference was presented as No. 6. 16 representative references with the strongest bursts (strength ≥ 4.95) in the group of references that started to burst at the same time (i.e., Nos. 1–3, 5–10, 12, and 14–19) and 7 representative references (i.e., Nos. 8, 10, and 19–23) with significant citation bursts in the past three years, which can be adopted to disclose the LTCI research trends and frontiers. Furthermore, we investigated the top five references in each cluster by cited counts. The higher the cited counts are, the more valuable and influential the paper is [24]. Table 4 lists detailed descriptions of 55 representative references.

Cluster #0 represents long-term care insurance, containing 120 references that were mostly published around 2015. The most active citer in this cluster was No. 10. Rhee’s [26] arguments received much attention, with results suggesting that it is important for middle-income countries to make some preparations before the LTCI program is implemented, such as a variety of benefit designs and the improvement of mechanisms of market competition and regulation. Brown and Wiener [35] proposed that the life care annuity provides a mechanism by which healthy individuals could purchase a policy on actuarially favorable terms and extends disability protection to the segment of the population that would not qualify for LTCI at retirement. Recently, there has also been interest in LTC financing [5,18] and influential factors of demand for LTCI, such as bequest motives [39] and insurance benefits [40].

The second largest cluster (#1) contained 105 references and was labeled as the Ohsaki cohort. As mentioned above, Tsuji is a representative scholar of the Ohsaki cohort. Cohort study is a type of epidemiological study in which subjects are selected based on a common condition or exposure, and are then observed for a subsequent outcome [28]. There are seven highly cited references in this cluster. Among them, two references adopted this method to examine the factors associated with disability in the elderly defined by the certification of eligibility for LTCI, such as social participation [28] and dental health status [43]. Furthermore, five references focused on strategies for implementing LTCI: strengthening collaborations between medical care and LTC [44], improving LTCI certification [3,45], and providing LTCI services for dementia [9,38]. Moreover, the silhouette value of the cluster is 0.953, indicating high consistency among the cited articles in this cluster, with the article of Satake et al. [3] being cited most often, by 18 articles.

Cluster #2, long-term care, ranked third in cluster size, including 104 references that were mostly published around 2010. LTC is an integration of a health and social system in which families, professionals, carers, and volunteers provide services to older people in need of care to improve their quality of life. Recent evidence has shown that the fast-growing demand for LTC places heavy burdens on family carers [33]. To address this, the Japanese government initiated mandatory public LTCI in 2000 [2,29], while in the USA, the Community Living Assistance Services and Supports Act was enacted to help pay for LTC services and support for disabled Americans [46]. Additionally, Brown et al. [15] concluded that factors such as preferences and beliefs, substitutes for insurance, substitutes for formal care, and features of the private market affected the demand for LTCI, and the policy interventions designed to address only one factor are unlikely to dramatically increase the demand for LTCI.

There are other clusters worth mentioning. References in cluster #3 have a common concern for the private LTCI market and found that the reasons for the small size of the private LTCI market were the crowding-out effect of the public Medicaid program [7,17,30,32] and asymmetric information in insurance markets [35]. The most active citer in cluster #4 [16,19,31,47,48] was Tsutsui [19,31], who focused on improving the LTCI system. Four references in cluster #5 reflected a common theme—the public LTCI model in Japan and in Germany [1,37,49,50]. References in cluster #6 were mainly concerned with a practical mix of public and private LTC financing [13,14,36,51,52]. Cluster #7, with a mean publication year of 2015, was dedicated to solving several problems of LTCI, such as expenditure growth [55], moral hazard [53], and potential market failures [54]. Meanwhile, the relationship between LTCI and the employment of informal caregivers has attracted a large amount of attention [8,11]. Cluster #8 was concerned with the guidelines of LTCI for reducing health cost [55], expanding LTCI coverage [56], and increasing LTCI benefits [57,58]. The common topic of cluster #9 was risky business in LTCI. In particular, adverse selection, the assessment of risk, and the information about the risk of nursing homes would have an impact on LTC financing [4,6,62], LTCI market size [60], and the welfare of the elderly [61].

Overall, the focus of these 10 clusters can be divided into 10 hot topics, namely, disability in the elderly, community-based service, LTC service, demand for and supply of LTCI, the LTCI market, risky business in LTCI, LTC financing, the LTCI model, policy of LTCI, and the LTCI system.

**Figure 7:**
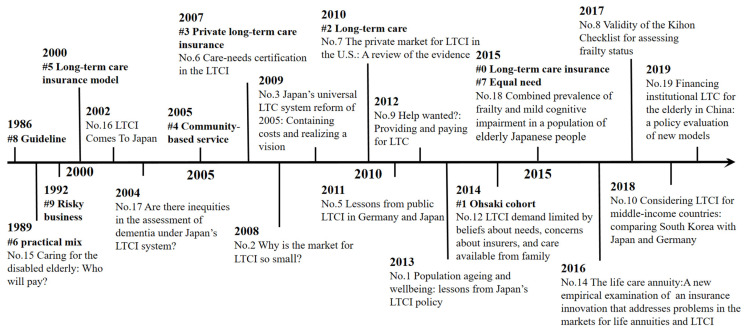
Timeline of the 10 largest clusters and 16 burst references. Note: # represents a knowledge cluster.

In order to get an impression of evolution of research fronts in LTCI research, 16 strong citation bursts (i.e., Nos. 1–3, 5–10, 12, 14–19) were drawn, as shown in Figure 7. An article with citation burst means it received special attention at a certain period of time. Furthermore, research clusters newly arising can be recognized as the new emerging trend which represented a thematic concentration in the bibliographic landscape. Therefore, the largest 10 emerging clusters were also considered to detect LTCI research frontiers. Figure 7 shows the timeline view of 10 clusters and 16 burst references with their respective research foci. The evolution trends of LTCI research in different periods were revealed as follows: in the early stage, before 2000, the research focused on the LTCI model and LTC financing; in the second stage, from 2001 to 2010, focus shifted to aspects of LTCI system establishment and the LTCI market, including public LTCI and private LTCI; in the third stage, from 2011 to 2021, the LTCI practice in various countries and the demand for LTCI received increased attention. In short, LTCI research trends have shifted from the supply side to the demand side, and from basic studies to practical applications.

#### 3.3.2. Keyword Co-Occurrence Analysis

**Figure 8:**
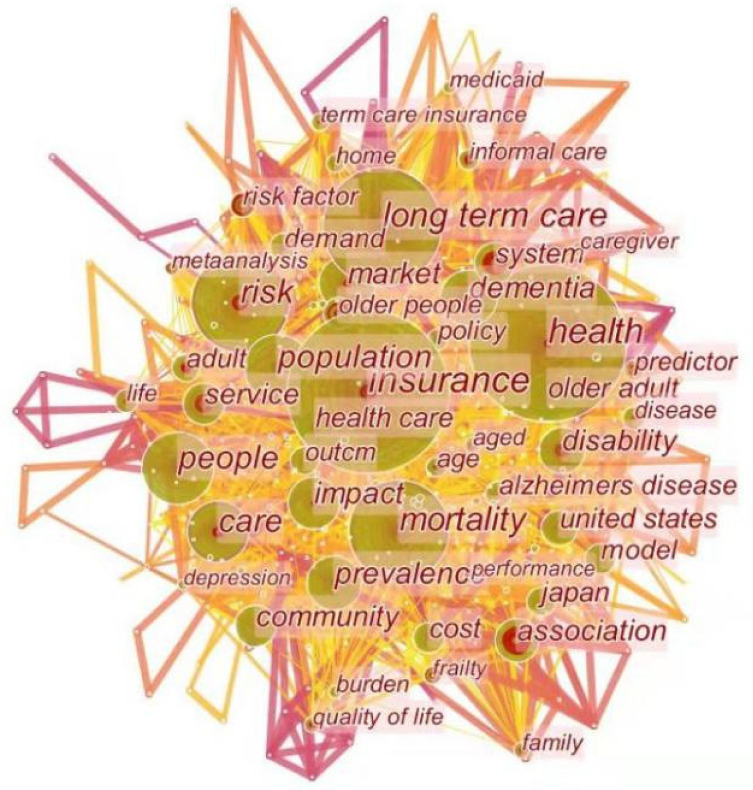
A visualization of the co-occurring keyword network.

Keywords are descriptive and significant words that present the core concepts and contents of research articles and show the development of a research field over time. The main features of the intellectual and landscape of LTCI keywords were three-fold: high-frequency keywords can reflect the research hotspots; keywords with high betweenness centrality represent major intellectual turning points connecting other keywords; and burst keywords represent new research frontiers. In order to further explore the hotspots and emerging trends of LTCI research, we produced a keyword co-occurrence network of articles with CiteSpace using both author keywords and the keywords plus. The parameters in CiteSpace remained the same, except for the node type being changed from “reference” to “keyword”. We merged similar keywords that were in fact the variants of the same entity, such as “long term care insurance”, “long-term care insurance”, and “LTCI”, were merged into “long-term care insurance (LTCI)”. Figure 8 shows the network of co-occurring keywords, with 552 nodes and 3976 links. The node size represents the frequency of the keyword in the record, lines that connect nodes are co-occurring links, and the colors of these lines show when a connection was made for the first time [23].

Similarly, we identified 34 representative keywords in terms of high betweenness centrality, high citation, and strong bursts. Table 5 lists the top 26 keywords with a co-occurrence frequency of more than 40 (i.e., Nos. 1–26). Among them, the five keywords “Health”, “Long-term care”, “Mortality”, “Risk”, and “Care”, from No. 2 to No. 6, had high betweenness centrality.

The top two keywords in terms of co-occurrence frequency were “insurance” (209) and “health” (198). Accordingly, the health status of the elderly is a hot topic in LTCI research. It is noteworthy that keywords such as “long-term care”, “mortality”, “risk”, “care”, and “health” have both high frequency and citation bursts. It is consistent with the fact that more efforts are devoted to these research themes which are pivotal in developing LTCI.

In essence, these 26 high-frequency keywords can be directly regarded as LTCI research hotspots, but they are so broad and macro that the overall understanding is poor. Therefore, Figure 8 is generated by integrating 26 high-frequency keywords and considering the co-occurring keywords. The emerging four main hot spots were as follows:

1. Disability in the elderly was extracted using keywords “health”, “people”, “dementia”, “prevalence”, “disability”, “older adult”, and “Alzheimer’s disease”. In the aging society and the age of longevity, older people are at a greater risk of disability [5,54]. This not only causes pain to the elderly but also seriously threatens their physical and mental health and daily activities, increasing the burden on families and society [4,6,48], and creates a greater demand for LTC [4,38]. Thus, as an innovative system to ensure the healthy development of the aging society, LTCI focuses on solving the problem of nursing for the disabled elderly, alleviating the pressure of family care and improving their quality of life [1,4,6,36].
2. Demand for and supply of LTCI was identified using the five keywords “long-term care”, “care”, “market”, “service”, and “demand”. The aging population presents LTCI with the challenge of increasing demand and insufficient supply. Lots of research proposed a host of potential explanations for the limited size of the LTCI market from two sides. On the demand side, several different factors, including the existence of Medicaid [15,18], the role of tax subsidies [7,54], bequest motives [35,54], individual characteristics [7,15], and cognitive factors [15,35], would have effects on the demand for LTCI. On the supply side, market function may be impaired by problems such as high transactions costs [7,15], imperfect competition [17,46], asymmetric information [7,30], or dynamic problems with long-term contracting [54].
3. LTC financing was extracted using the keywords “long-term care”, “risk”, and “cost”. The current debate about LTC financing mainly focuses on the choice of public and private financing strategies. Furthermore, escalating costs, a bias in public financing toward institutional care and access problems led to an increasing demand for reform of LTC financing [63]. Many suggestions were put forward to solve the problem, such as enhanced Medicaid [61], improved consumer education and publicity [62], tax law changes [18,54], and solving the coordination problems of different financing regimes [12]. Although these proposals have different emphases, they have reached a consensus on the degree of public–private mix in financing that would be much more feasible in LTC [10].
4. The LTCI system comprised five representative keywords, namely, “system”, “impact”, “United States”, “Japan”, and “model”. Since the 1970s, various countries have established LTCI systems, which achieved remarkable results in effectively responding to the growing needs of the elderly for LTC, addressing newly developing social risks and promoting social justice [29,44,53,62]. Overall, the practice of LTCI in developed countries formed two models [22]: (1) public LTCI represented by Germany, Japan, and South Korea [2,3,26]; (2) private LTCI, with the United States as the typical representative [17,30,32]. These two models have some differences in insurance coverage, financing, payments, and service contents [4,10,30].

Moreover, the keywords showing occurrence bursts within the past three years (2019–2021) were frailty (3.8, 2019–2021), quality (3.37, 2019–2021), institutionalization (2.27, 2019–2021), consequence (2.05, 2019–2021), mobility (1.83, 2019–2021), fall (1.79, 2019–2021), expectation (1.35, 2019–2021), diagnosis (1.11, 2019–2021), access (1.06, 2019–2021), and coverage (0.12, 2019–2021). These findings indicated that much research attention was directed to these areas. On the whole, we found that the emerging LTCI research frontiers could be summarized as follows: frailty in the elderly, demand for LTCI, and the LTCI system.

## 4. Conclusions

In this paper, we drew on bibliometric data relating to 1568 journal articles listed on the WoSCC database. The scientific output and citations of LTCI research and the collaboration networks were visualized to examine the current status, development, and major contributors of the research. Keyword co-occurrence analysis and document co-citation analysis enabled us to explore hot topics, evolution trends, and new frontiers in LTCI research. The specific findings were as follows:

Firstly, LTCI research has developed steadily, and this trend will continue. The annual publication in the LTCI research field showed a noticeable upward trend in recent years, and it is estimated to reach 142 in 2022 and 125 in 2023, confirming that LTCI research is likely to attract increasing attention in the next few years. The polynomial trendline of citations indicated an explosive growth, especially in 2016–2021, suggesting that the field has experienced a period of rapid development, especially since the end of 2019 when the COVID-19 pandemic started. COVID-19 has severely impaired the physical and mental health of elderly people, creating an increased need for LTCI. Therefore, scholars in different disciplines should better recognize the importance of LTCI and investigate more relevant issues to it.

Secondly, although the research output of the major contributors is abundant, the international collaboration among them should be strengthened. Japan, the USA, and Germany are the three leading countries in LTCI research, forming a leading research group. Due to the large elderly population in these countries, they had to start the exploration of LTCI systems earlier. The degree of LTCI development and the wider insurance coverage contributed to the prominent position of these countries. However, since these three countries developed different LTCI models, the collaborative relationships among them were not strong. Furthermore, the financing methods, insurance coverage, and service provided by LTCI vary greatly from one country to another, increasing the difficulty of cooperation between them. Nevertheless, globalization requires cooperation and calls for research from multiple perspectives. Therefore, a close international cooperation network is urgently needed. The top 15 most productive institutions mostly came from Japan and the USA, which proved to be the main research powers in this field. Tokyo University was identified as the most influential institution in this field based on the publication counts and betweenness centrality, and Tsuji was the most productive and high-impact author. These contributors provided a wealth of results for LTCI research with great significance. By contrast, developing countries were under-represented in the global research network. We noted that although these countries started late in exploring LTCI, institutions from these countries have accelerated their efforts to participate in LTCI research. On the basis of the experience of the developed countries, they explored the LTCI system suitable for their own countries. It is suggested that developing countries still have strong development potential in this field.

Thirdly, with the LTCI research development, future research will focus on solving practical problems and improving the LTCI systems. Consistent results were found from the current analysis of the results of visualizing the intellectual landscape of references and keywords. It was revealed that the common LTCI research hot topics in the 1984–2021 period were disability in the elderly, community-based service, LTC service, demand for and supply of LTCI, the LTCI market, risky business in LTCI, LTC financing, the LTCI model, policy of LTCI, and the LTCI system. The research on these hot topics examined LTCI from the perspectives of health and care, economics and finance, health policy services, sociology, demography and psychology, and so on. The LTCI evolution trends showed three stages: an early stage (before 2000), where research was primarily focused on the LTCI model and financing LTC in both public and private ways; a second stage (2001–2010), where the focus shifted to aspects of the LTCI system establishment and the LTCI market, including public LTCI and private LTCI; and a third stage (2011–2021), where LTCI practice in various countries and the demand for LTCI received considerable attention. It was reflected that the trend of LTCI research had shifted from the supply side to the demand side, and from basic studies to practical applications. In summary, the research has become substantially extensive and in-depth. From the research results, we observe that many countries and regions have taken the establishment of LTCI systems as an important way to deal with the aging crisis. In order to solve the problem of elderly care, some developed countries gradually formed an elderly care model with LTCI as the main body and financial and tax support as the auxiliary through the development of an LTCI system, establishing a multi-channel financing mechanism, increasing the nursing level and the payment standard, and improving the nursing service system. According to the analysis, the common research frontiers were frailty in the elderly, demand for and supply of LTCI, and the LTCI system. Moreover, optimal mix of public and private funding, interaction with other parts of welfare such as pension schemes, impact of culture on formal and informal care, role of taxes or premium subsidies, and the impact of COVID-19 on the market and the system of LTCI also seem to be promising research directions.

In conclusion, this paper provides valuable information to LTCI researchers to identify new perspectives concerning major countries/regions, institutions, researchers, hot topics, evolution trends, and new research frontiers. Moreover, for LTCI practitioners, this study presents accurate information regarding the key authors and institutions best suited to assist in developing LTCI systems. This research provides a new scientific visualization method to explore the status and direction of LTCI development. However, there was also a limitation in terms of our scientometric analysis. We only collected bibliographic records on LTCI from one database—the WoSCC. Future studies may carry out a broader study based on other databases to complement the preliminary results with the current study. While this study is a good starting point for reviewing the literature on LTCI, researchers who want to delve into the field should review the searched literature from different perspectives to conduct more in-depth research. In addition, the retrieval strategy “long-term care” and “insurance” could be improved by using different search terms other and connecting them with a Boolean operation.

## Figures and Tables

**Table 1:** Top 15 countries/regions based on publications and centrality.

Rank	Country/Region	Count	Centrality	Rank	Country/Region	Centrality	Count
1	Japan	491	0.24	1	USA	0.39	430
2	USA	430	0.39	2	Japan	0.24	491
3	Germany	201	0.15	3	China	0.24	68
4	South Korea	107	0.00	4	England	0.22	61
5	China	68	0.24	5	Belgium	0.19	32
6	England	61	0.22	6	Germany	0.15	201
7	France	51	0.05	7	Netherlands	0.13	32
8	Canada	49	0.00	8	Israel	0.06	23
9	Taiwan	45	0.01	9	Italy	0.06	20
10	Spain	34	0.03	10	France	0.05	51
11	Belgium	32	0.19	11	Spain	0.03	34
12	Australia	32	0.03	12	Australia	0.03	32
13	Netherlands	32	0.13	13	Sweden	0.02	5
14	Switzerland	27	0.01	14	Taiwan	0.01	45
15	Israel	23	0.06	15	Switzerland	0.01	27

**Table 2:** Top 12 productive institutions.

Rank	Institution	Count	Centrality	Country
1	University of Tokyo	78	0.13	Japan
2	National Center for Geriatrics and Gerontology	73	0.07	Japan
3	Tohoku University	64	0.03	Japan
4	University of Tsukuba	43	0.04	Japan
5	Harvard University	31	0.07	USA
6	Tokyo Metropolitan	30	0.02	Japan
7	Kyoto University	30	0.01	Japan
8	Chiba University	29	0.00	Japan
9	National Institute of Public Health	21	0.02	Japan
10	Keio University	19	0.02	Japan
11	Osaka University	19	0.02	Japan
12	University of Liège	18	0.01	Belgium

**Table 3:** Top five most cited articles from Tsuji.

Author	Title of Articles	Year	Count
Ichiro Tsuji (47, Tohoku University)	Green tea consumption and the risk of incident functional disability in elderly Japanese: The Ohsaki Cohort 2006 Study	2012	49
	The Ohsaki Cohort 2006 Study: Design of Study and Profile of Participants at Baseline	2010	48
	Green Tea Consumption and the Risk of Incident Dementia in Elderly Japanese: The Ohsaki Cohort 2006 Study	2016	39
	Dietary Patterns and Incident Functional Disability in Elderly Japanese: The Ohsaki Cohort 2006 Study	2014	39
	Long-term impact of the 2011 Great East Japan Earthquake and tsunami on functional disability among older people: A 3-year longitudinal comparison of disability prevalence among Japanese municipalities	2015	30

**Table 4:** List of 55 representative references based on citations, centrality, and bursts.

No.	Count	Centrality	Strength	Reference	Year	Begin	End	Cluster ID
1	25	0.04	12.38	Tamiya et al. [29]	2011	2013	2016	#2
2	24	0.07	12.42	Brown and Finkelstein [17]	2007	2008	2012	#3
3	24	0.02	12.97	Tsutsui and Muramatsu [19]	2007	2009	2012	#4
4	22	0.01	11.12	Brown and Finkelstein [30]	2008	2009	2013	#3
5	20	0.02	9.37	Campbell et al. [2]	2010	2011	2015	#2
6	19	0.11	11.12	Tsutsui and Muramatsu [31]	2005	2007	2010	#4
7	19	0.04	9.24	Brown and Finkelstein [32]	2009	2010	2014	#3
8	18	0.03	6.63	Satake et al. [3]	2016	2017	2021	#1
9	17	0.08	7.38	OECD [33]	2011	2012	2016	#2
10	16	0.01	7.86	Rhee [26]	2015	2018	2021	#0
11	16	0.02	9.19	Finkelstein and McGarry [34]	2006	2008	2011	#3
12	16	0.03	6.03	Brown et al. [15]	2012	2014	2017	#2
13	14	0.02	6.07	Kanamori et al. [28]	2014	2016	2019	#1
14	13	0.01	6.63	Brown and Warshawsky [35]	2013	2016	2018	#0
15	13	0.00	8.14	Rivlin and Wiener [36]	1988	1989	1993	#6
16	10	0.06	6.24	Campbell and Ikegami [37]	2000	2002	2005	#5
17	8	0.03	4.79	Arai et al. [16]	2003	2004	2008	#4
18	10	0.01	5.12	Shimada et al. [38]	2013	2015	2017	#1
19	9	0.02	4.95	Yang et al. [5]	2016	2019	2021	#0
20	8	0.00	4.4	Lockwood [39]	2018	2019	2021	#0
21	8	0.01	4.4	Maarseand and Jeurissen [12]	2016	2018	2021	#7
22	8	0.00	4.4	Klimaviciate [40]	2017	2019	2021	#0
23	8	0.01	3.91	Livingston et al. [9]	2017	2019	2021	#1
24	11	0.01	4.97	Finkelstein et al. [41]	2013	2014	2017	#0
25	10	0.01	4.97	Fong et al. [42]	2015	2017	2019	#0
26	10	0.04	0.05	Costa-Font et al. [18]	2015	2015	2019	#0
27	11	0.01	6.27	Yamamoto et al. [43]	2012	2012	2017	#1
28	11	0.01	4.77	Moriyama et al. [44]	2014	2016	2019	#1
29	11	0.02	4.13	Fukutomi et al. [45]	2015	2015	2019	#1
30	11	0.01	5.78	Brown and Finkelstein [46]	2011	2014	2016	#2
31	9	0.01	4.83	Brown et al. [7]	2007	2009	2012	#3
32	8	0.06	4.53	Ikegami [47]	2007	2010	2012	#4
33	5	0.06	0.05	Kato et al. [48]	2009	2009	2012	#4
34	7	0.05	4.36	Cuellar and Wiener [1]	2000	2002	2005	#5
35	6	0.03	0.05	Ikegami and Campbell [10]	2002	2002	2006	#5
36	5	0.03	0.05	Campbell and Ikegami [49]	2003	2004	2006	#5
37	4	0.01	0.05	Ikegami [50]	1997	1999	2001	#5
38	4	0.00	0.05	Vangelder et al. [51]	1991	1991	1993	#6
39	4	0.00	0.05	Spence and Wiener [13]	1990	1991	1992	#6
40	4	0.00	0.05	Rice [14]	1989	1990	1991	#6
41	4	0.00	0.05	Liu et al. [52]	1990	1991	1992	#6
42	9	0.02	0.05	Bakx et al. [53]	2015	2015	2019	#7
43	6	0.00	0.05	Fu et al. [11]	2017	2019	2020	#7
44	5	0.00	0.05	Van et al. [8]	2013	2014	2017	#7
45	5	0.00	0.05	Norton [54]	2016	2019	2019	#7
46	2	0.00	0.05	Bowen [55]	1986	1986	1988	#8
47	2	0.00	0.05	Barker [56]	1987	1987	1990	#8
48	1	0.00	0.05	Cafferata [57]	1985	1988	1988	#8
49	1	0.00	0.05	Kane et al. [58]	1985	1986	1986	#8
50	1	0.00	0.05	Katz et al. [59]	1983	1986	1986	#8
51	5	0.00	0.05	Cohen et al. [60]	1993	1994	1996	#9
52	2	0.00	0.05	Scanlon [6]	1992	1995	1995	#9
53	2	0.00	0.05	Newhouse [61]	1992	1996	1996	#9
54	2	0.00	0.05	Murtaugh et al. [62]	1990	1994	1994	#9
55	2	0.00	0.05	Liu et al. [4]	1990	1994	1994	#9

Note: # represents a knowledge cluster.

**Table 5:** List of 26 representative keywords based on occurrences and centrality.

No.	Count	Centrality	Keyword	No.	Count	Centrality	Keyword
1	209	0.09	Insurance	14	66	0.03	Association
2	198	0.10	Health	15	62	0.09	Service
3	160	0.20	Long-term care	16	61	0.05	Community
4	128	0.14	Mortality	17	59	0.04	Older adult
5	127	0.13	Risk	18	59	0.04	System
6	101	0.13	Care	19	57	0.07	Healthcare
7	94	0.07	Population	20	56	0.06	United States
8	93	0.03	People	21	52	0.09	Cost
9	85	0.05	Market	22	50	0.04	Japan
10	78	0.05	Dementia	23	48	0.04	Model
11	78	0.03	Prevalence	24	48	0.04	Adult
12	74	0.09	Impact	25	47	0.04	Demand
13	69	0.07	Disability	26	43	0.04	Alzheimer’s disease

## Data Availability

Not applicable.

## References

[1] Cuellar A.E., Wiener J.M. (2000). Can Social Insurance for Long-Term Care Work? The Experience of Germany: Germany may be the only country in which most of the beneficiaries and the money are in community-based long-term care settings. Health Aff..

[2] Campbell J.C., Ikegami N., Gibson M.J. (2010). Lessons from public long-term care insurance in Germany and Japan. Health Aff..

[3] Satake S., Senda K., Hong Y.J., Miura H., Endo H., Sakurai T., Kondo I., Toba K. (2016). Validity of the Kihon Checklist for assessing frailty status. Geriatr. Gerontol. Int..

[4] Liu K., Manton K.G., Liu B.M. (1990). Morbidity, disability, and long-term care of the elderly: Implications for insurance financing. Milbank Q..

[5] Yang W., Jingwei He A., Fang L., Mossialos E. (2016). Financing institutional long-term care for the elderly in China: A policy evaluation of new models. Health Policy Plan..

[6] Scanlon W.J. (1992). Possible reforms for financing long-term care. J. Econ. Perspect..

[7] Brown J.R., Coe N.B., Finkelstein A. (2007). Medicaid crowd-out of private long-term care insurance demand: Evidence from the health and retirement survey. Tax Policy Econ..

[8] Van Houtven C.H., Coe N.B., Skira M.M. (2013). The effect of informal care on work and wages. J. Health Econ..

[9] Livingston G., Sommerlad A., Orgeta V., Costafreda S.G., Huntley J., Ames D., Ballard C., Banerjee S., Burns A., Cohen-Mansfield J. (2017). Dementia prevention, intervention, and care. Lancet.

[10] Ikegami N., Campbell J.C. (2002). Choices, policy logics and problems in the design of long–term care systems. Soc. Policy Adm..

[11] Fu R., Noguchi H., Kawamura A., Takahashi H., Tamiya N. (2017). Spillover effect of Japanese long-term care insurance as an employment promotion policy for family caregivers. J. Health Econ..

[12] Maarse J.H., Jeurissen P.P. (2016). The policy and politics of the 2015 long-term care reform in the Netherlands. Health Policy.

[13] Spence D.A., Wiener J.M. (1990). Estimating the extent of Medicaid spend-down in nursing homes. J. Health Polit. Policy Law.

[14] Rice T. (1989). The use, cost, and economic burden of nursing-home care in 1985. Med. Care.

[15] Brown J.R., Goda G.S., McGarry K. (2012). Long-term care insurance demand limited by beliefs about needs, concerns about insurers, and care available from family. Health Aff..

[16] Arai Y., Zarit S.H., Kumamoto K., Takeda A. (2003). Are there inequities in the assessment of dementia under Japan’s LTC insurance system?. Int. J. Geriatr. Psychiatry.

[17] Brown J.R., Finkelstein A. (2007). Why is the market for long-term care insurance so small?. J. Public Econ..

[18] Costa-font J., Courbage C., Swartz K. (2015). Financing long-term care: Ex ante, ex post or both?. Health Econ..

[19] Tsutsui T., Muramatsu N. (2007). Japan’s universal long-term care system reform of 2005: Containing costs and realizing a vision. J. Am. Geriatr. Soc..

[20] Houde S.C., Gautam R., Kai I. (2007). Long-term care insurance in Japan: Implications for US long-term care policy. J. Gerontol. Nurs..

[21] Eling M., Ghavibazoo O. (2019). Research on long-term care insurance: Status quo and directions for future research. Geneva Pap. Risk Insur. Issues Pract..

[22] Chen L., Zhang L., Xu X. (2020). Review of evolution of the public long-term care insurance (LTCI) system in different countries: Influence and challenge. BMC Health Serv. Res..

[23] Chen C.M., Dubin R., Kim M.C. (2014). Orphan drugs and rare diseases: A scientometric review (2000–2014). Expert Opin. Orphan Drugs.

[24] Chen C.M. (2006). CiteSpace II: Detecting and visualizing emerging trends and transient patterns in scientific literature. J. Am. Soc. Inf. Sci. Technol..

[25] Fu L., Sun Z., He L., Liu F., Jing X. (2019). Global long-term care research: A scientometric review. Int. J. Environ. Res. Public Health.

[26] Rhee J.C., Done N., Anderson G.F. (2015). Considering long-term care insurance for middle-income countries: Comparing South Korea with Japan and Germany. Health Policy.

[27] Courbage C., Roudaut N. (2011). Long-term care insurance: The French example. Eur. Geriatr. Med..

[28] Kanamori S., Kai Y., Aida J., Kondo K., Kawachi I., Hirai H., Shirai K., Ishikawa Y., Suzuki K., JAGES Group (2014). Social participation and the prevention of functional disability in older Japanese: The JAGES cohort study. PLoS ONE.

[29] Tamiya N., Noguchi H., Nishi A., Reich M.R., Ikegami N., Hashimoto H., Shibuya K., Kawachi I., Campbell J.C. (2011). Population ageing and wellbeing: Lessons from Japan’s long-term care insurance policy. Lancet.

[30] Brown J.R., Finkelstein A. (2008). The interaction of public and private insurance: Medicaid and the long-term care insurance market. Am. Econ. Rev..

[31] Tsutsui T., Muramatsu N. (2005). Care-needs certification in the long-term care insurance system of Japan. J. Am. Geriatr. Soc..

[32] Brown J.R., Finkelstein A. (2009). The Private Market for Long-Term Care Insurance in the United States: A Review of the Evidence. J. Risk Insur..

[33] Organization for Economic Co-operation and Development (OECD) (2011). Help Wanted? Providing and Paying for Long-Term Care.

[34] Finkelstein A., McGarry K. (2006). Multiple dimensions of private information: Evidence from the long-term care insurance market. Am. Econ. Rev..

[35] Brown J., Warshawsky M. (2013). The life care annuity: A new empirical examination of an insurance innovation that addresses problems in the markets for life annuities and long-term care insurance. J. Risk Insur..

[36] Rivlin A.M., Wiener J.M. (1991). Caring for the Disabled Elderly: Who Will Pay? Washington: Brookings Institution. Health Serv. Res..

[37] Campbell J.C., Ikegami N. (2000). Long-Term Care Insurance Comes to Japan: A major departure for Japan, this new program aims to be a comprehensive solution to the problem of caring for frail older people. Health Aff..

[38] Shimada H., Makizako H., Doi T., Yoshida D., Tsutsumimoto K., Anan Y., Uemura K., Ito T., Lee S., Park H. (2013). Combined prevalence of frailty and mild cognitive impairment in a population of elderly Japanese people. J. Am. Med. Dir. Assoc..

[39] Lockwood L.M. (2018). Incidental bequests and the choice to self-insure late-life risks. Am. Econ. Rev..

[40] Klimaviciute J. (2017). Long-term care insurance and intra-family moral hazard: Fixed vs proportional insurance benefits. Geneva Risk Insur. Rev..

[41] Finkelstein A., Luttmer E.F., Notowidigdo M.J. (2013). What good is wealth without health? The effect of health on the marginal utility of consumption. J. Eur. Econ. Assoc..

[42] Fong J.H., Shao A.W., Sherris M. (2015). Multistate actuarial models of functional disability. N. Am. Actuar. J..

[43] Yamamoto T., Kondo K., Hirai H., Nakade M., Aida J., Hirata Y. (2012). Association between self-reported dental health status and onset of dementia: A 4-year prospective cohort study of older Japanese adults from the Aichi Gerontological Evaluation Study (AGES) Project. Psychosom. Med..

[44] Moriyama Y., Tamiya N., Kamimura A., Sandoval F., Luptak M. (2014). Doctors’ opinion papers in long-term care need certification in Japan: Comparison between clinic and advanced treatment hospital settings. Public Policy Adm. Res..

[45] Fukutomi E., Okumiya K., Wada T., Sakamoto R., Ishimoto Y., Kimura Y., Chen W., Imai H., Kasahara Y., Fujisawa M. (2015). Relationships between each category of 25-item frailty risk assessment (Kihon Checklist) and newly certified older adults under Long-Term Care Insurance: A 24-month follow-up study in a rural community in Japan. Geriatr. Gerontol. Int..

[46] Brown J.R., Finkelstein A. (2011). Insuring long-term care in the United States. J. Econ. Perspect..

[47] Ikegami N. (2007). Rationale, design and sustainability of long-term care insurance in Japan-in retrospect. Soc. Policy Soc..

[48] Kato G., Tamiya N., Kashiwagi M., Sato M., Takahashi H. (2009). Relationship between home care service use and changes in the care needs level of Japanese elderly. BMC Geriatr..

[49] Campbell J.C., Ikegami N. (2003). Japan’s radical reform of long-term care. Soc. Policy Adm..

[50] Ikegami N. (1997). Public long-term care insurance in Japan. JAMA.

[51] Van Gelder S., Johnson D. (1991). Long-Term Care Insurance: A Market Update.

[52] Liu K., Doty P., Manton K. (1990). Medicaid spenddown in nursing homes. Gerontologist.

[53] Bakx P., Chernichovsky D., Paolucci F., Schokkaert E., Trottmann M., Wasem J., Schut F. (2015). Demand-side strategies to deal with moral hazard in public insurance for long-term care. J. Health Serv. Res. Policy.

[54] Norton E.C., Piggott J., Woodland A. (2016). Health and long-term care. Handbook of the Economics of Population Aging.

[55] Bowen O.R. (1986). Catatrophic Illness Expenses: Report to the President.

[56] Barker W.H. (1987). Adding Life to Years: Organized Geriatric Services in Great Britain and Implications for the United States.

[57] Cafferata G.L. (1985). Private health insurance of the Medicare population and the Baucus legislation. Med. Care.

[58] Kane R.A., Kane R.L. (1985). The Feasibility of Universal Long-Term-Care Benefits: Ideas from Canada. N. Engl. J. Med..

[59] Katz S., Branch L.G., Branson M.H., Papsidero J.A., Beck J.C., Greer D.S. (1983). Active life expectancy. N. Engl. J. Med..

[60] Cohen M.A., Kumar N., Wallack S.S. (1993). New Perspectives on the Afford ability of Long-term Care Insurance and Potential Market Size. Gerontologist.

[61] Newhouse J.P. (1992). Medical care costs: How much welfare loss?. J. Econ. Perspect..

[62] Murtaugh C.M., Kemper P., Spillman B.C. (1990). The risk of nursing home use in later life. Med. Care.

[63] Cohen M.A., Kumar N., McGuire T., Wallack S.S. (1992). Financing long-term care: A practical mix of public and private. J. Health Polit. Policy Law.

